# Laparoscopic Versus Open Ventral Hernia Repair: Experience at a Tertiary Care Center in Western Rajasthan

**DOI:** 10.7759/cureus.27279

**Published:** 2022-07-26

**Authors:** Murlidhar Kalyan, Shaitan Singh Rathore, Vijay Verma, Sarthak Sharma, Mahendra Choudhary, Irshad Irshad, Pradeep Gupta

**Affiliations:** 1 General Surgery, Dr. Sampurnanand Medical College, Jodhpur, IND

**Keywords:** laparoscopic vs open ventral hernia repair, visual analog scale (vas), mesh repair, ipom, open ventral hernia repair, laparoscopic ventral hernia repair

## Abstract

Background

Ventral hernias are the second most common type of hernias accounting for 21-35% of all hernia types. Ventral hernia includes incisional, umbilical, epigastric, and Spigelian hernias, among others. Currently, patients and surgeons prefer laparoscopic repair of ventral hernias. This study aimed to compare laparoscopic with open hernia repair in terms of various operative and patient parameters.

Methodology

This was a prospective observational study conducted in the Department of General Surgery, Dr. Sampurnanand Medical College, Jodhpur. All patients admitted with a diagnosis of ventral hernia for mesh repair (open as well as laparoscopic) were included. Laparoscopic and open hernia repair were compared in terms of operative time, postoperative hospital stay, time to resume routine activity, postoperative complications, and recurrence.

Results

Among the 50 patients included in the study (25 patients each in the laparoscopic and open groups), the mean operative time was 57.52 ± 5.80 minutes in the laparoscopic group and 59.8 ± 11.15 minutes in the open group. The mean hospital stay in the laparoscopy and open groups was 7.4 ± 1.58 days and 9.88 ± 2.96 days, respectively (p-value = 0.0006; significant). Postoperative pain (using the visual analog scale score) was less in patients who underwent laparoscopic surgery (p-value = 0.001; significant). Seroma and surgical site infections were the most common complications which were observed more in the open hernia repair group. Recurrence was seen in one case operated by the open technique.

Conclusions

Laparoscopic ventral hernia repair is technically safer, effective, and feasible with better clinical outcomes in patients seeking treatment in a government hospital.

## Introduction

A ventral hernia occurs due to a weakness in the musculofascial layer of the anterior abdominal wall [1]. Ventral hernia includes paraumbilical, epigastric, Spigelian, and incisional hernias [2,3]. These hernias mainly present as swelling and rarely result in complications, such as strangulation and incarceration, and present with respective manifestations. Commonly, hernias are clinically diagnosed and do not require any special investigations [4]. Hernia repair can be carried out as an open repair or a laparoscopic repair.

Increased range of wound infection and wound-related complications in open hernia repair has led to continuing research for the optimal method of management of ventral hernia and has led surgeons to accept the laparoscopic approach [5]. The open repair requires adequate subcutaneous resection, raising of flaps, and drain insertion, along with increased wound complications [6].

The recent introduction of laparoscopic surgery for ventral hernias is gaining popularity and is being practiced by surgeons. The laparoscopic procedure helps the surgeon recognize the margins of the defect and identify any missed defects in clinical evaluation. The laparoscopic procedure enables the identification and management of occult hernias [7].

This study aimed to compare laparoscopic and open hernioplasty in uncomplicated ventral hernia concerning operative data, postoperative pain, postoperative complications, hospital stay, time to return to routine activity, and recurrence.

## Materials and methods

This study was conducted in the Mathura Das Mathur Hospital affiliated with Dr. Sampuranand Medical College, Jodhpur, Rajasthan. The Institutional Ethics Committee of Dr. Sampuranand Medical College, Jodhpur, issued approval SNMC/IEC/455 before the initiation of the study and after obtaining written and informed consent from the patients. This was a prospective observational study. Inclusion and exclusion criteria are depicted in Table 1.

**Table 1:** Inclusion and exclusion criteria.

Inclusion criteria	Exclusion criteria
All patients admitted to surgical units with a ventral hernia for mesh repair (open as well as laparoscopic)	Non-midline hernias
	Hernia after cesarean section
	Hernia after open appendicectomy
	Spigelian hernia
	Lumbar hernia
	Complicated hernia

Study objectives

In this study, we aimed to establish the differences in laparoscopic and open hernia surgery in terms of (1) operative time and postoperative hospital stay; (2) time to resume routine activity and work; (3) postoperative complications; and (4) recurrence.

Methodology

This study included 50 patients after applying the inclusion and exclusion criteria. The patients were distributed into either of the two groups (laparoscopic or open) based on the patient’s preferences. All patients were evaluated by obtaining a proper history and performing a detailed physical examination and routine blood investigations. All patients underwent a preanaesthetic checkup (PAC) before surgery. Preoperative preparation included intravenous (IV) fluids to correct dehydration and electrolyte imbalances. Appropriate IV antibiotics were administered as prophylaxis. Patients were subjected to either of the two procedures, namely, laparoscopic hernia repair (intraperitoneal onlay mesh repair, IPOM) or open hernia repair with mesh placement (open hernioplasty).

Procedure for Open Ventral Hernia Repair

The onlay technique was used for open repair. The skin was incised, the hernia sac was dissected and opened, the adherent viscera was freed, and the contents were reduced. Subcutaneous dissection was done around the defect. The fascial defect was closed with interrupted polypropylene 1 suture. Onlay polypropylene mesh was fixed to the edges of the defect with an overlap of 5 cm in all directions with interrupted Prolene (2-0) sutures. Suction drain size 14 was placed in the subcutaneous plane and the closure was done.

Procedure for Laparoscopic Ventral Hernia Repair

A pneumoperitoneum was created after inserting a Veress needle at Palmer’s point with carbon dioxide gas up to 12 mmHg pressure. One 10 mm port was inserted (Camera port), and three 5 mm ports (working port) were inserted at variable sites depending upon the location of the hernia. The defect was identified and measured using a metric scale. Mesh of a suitable size with 5 cm overlap around the margins was inserted via a 10 mm port and fixed to the anterior abdominal wall with absorbable tacks. The sheath was closed with vicryl port suture, and the skin was closed with Ethilon 3-0 reverse cutting suture. A pressure dressing was applied over the defect.

Postoperative Assessment

The visual analog scale (VAS) was used to assess pain in the postoperative period, and appropriate analgesia in the form of intramuscular (IM) injection of non-steroidal anti-inflammatory drugs (NSAIDs) was given until the resumption of oral feed. Any complications such as seroma, hematoma, flap necrosis, and infection were noted in the postoperative period and adequately managed.

Follow-up

Postoperatively, patients were motivated that they return to their daily routine activity as early as possible. Patients were re-evaluated at one month, three months, and six months. Surgical wounds were evaluated for seroma, infection, and wound dehiscence. The long-term evaluation included persisting pain at the surgical site, daily routine activity, and recurrence if any.

Statistical analysis

SPSS version 15.0 (SPSS Inc., Chicago, IL, USA) was used for data analysis. Mean ± standard deviation (SD) was used for continuous variables, and number (%) was used for categorical variables. The chi-square/Fisher exact test was used to determine the significance of the study parameters. A p-value of <0.05 was considered significant.

## Results

In the present study, 50 patients were included. In total, 25 patients underwent laparoscopic hernia repair (IPOM) and 25 patients underwent open hernia repair (onlay hernioplasty). The youngest patient was 24 years old and the oldest was 76 years old. The mean age at presentation was 51.6 ± 12.37 years. In our study, the highest number of cases were noted between 41 and 60 years of age. The mean age of patients who underwent laparoscopic and open hernia repair was 52.29 ± 12.96 years and 50.78 ± 11.88 years, respectively. Overall, 54% of patients were males while 46% of patients were females. The male-to-female ratio was 1:1.08 and 1.5:1 in the laparoscopic and open hernia repair groups, respectively. Swelling was the most consistent chief complaint, present in 100% of cases. In total, 44% of cases presented with pain along with swelling. The clinical characteristics of patients are depicted in Table 2.

**Table 2:** Demographic profile and clinical features of the patients.

	Laparoscopic repair group (n = 25)	Open repair group (n = 25)
Gender distribution		
Males	12 (48%)	15 (60%)
Females	13 (52%)	10 (40%)
Mean age (years)	52.29 ± 12.96	50.78 ± 11.88
Chief complaints		
Swelling	25 (100%)	25 (100%)
Pain	10 (40%)	12 (48%)
Vomiting	0	3 (12%)

The most common diagnosis was paraumbilical hernia (34%), followed by epigastric (22%), umbilical hernia (22%), and incisional hernia (20%) (Table 3).

**Table 3:** Distribution according to the type of hernia. *One patient had both umbilical and incisional hernia.

Types of hernia	Laparoscopic group (n = 25)	Open group (n = 25)
Paraumbilical	11 (44%)	6 (24%)
Umbilical	8 (32%)	3 (12%)
Epigastric	3 (12%)	8 (32%)
Incisional	2 (8%)	8 (32%)
Umbilical with incisional*	1 (4%)	0 (0%)

The most common comorbidity was hypertension and type 2 diabetes mellitus (Table 4).

**Table 4:** Distribution of comorbidities. DM: diabetes mellitus; CAD: coronary artery disease; COPD: chronic obstructive pulmonary disease

Comorbidities	Laparoscopic group (n = 25)	Open group (n = 25)
Type 2 DM	5 (20%)	4 (16%)
Hypertension	4 (16%)	5 (20%)
Hypothyroidism	3 (12%)	1 (4%)
CAD	2 (8%)	Nil
COPD	1 (4%)	4 (16%)

Overall, 60% of cases had omentum as the content of the hernia sac, and 80% and 40% of patients had omentum as the content in the laparoscopic and open surgery groups, respectively. Moreover, 54% of patients had an abdominal wall defect size of <3 cm, followed by 42% having defect size between 3 and 6 cm. The mean defect size was 2.54 ± 0.73 cm and 3.42 ± 1.81 cm in the laparoscopic and open hernia groups, respectively.

The mean operative time was 57.52 ± 5.80 minutes in the laparoscopic surgery group and 59.8 ± 11.15 minutes in the open surgery group (p-value = 0.369).

Postoperative pain was less in patients who underwent laparoscopic surgery (p-value = 0.001; significant), as depicted in Table 5.

**Table 5:** VAS score for postoperative pain assessment. *P-value of <0.05 is statistically significant; #chi-square test. VAS: visual analog scale

VAS Score	Laparoscopic group (n = 25)	Open group (n=25)	P-value
≤3	21 (84%)	9 (36%)	0.001*#
4-5	4 (16%)	9 (36%)	
≥6	0	7 (28%)	

Further, 56% and 64% of cases were discharged between the fifth and eighth postoperative days in the laparoscopic surgery and open surgery groups, respectively. However, 0% and 24% of patients had a postoperative stay of ≥nine days (p-value = 0.013; significant).

The hospital stay was shorter in the laparoscopy group than in the open group. The mean hospital stay in the laparoscopy and open groups was 7.4 ± 1.58 days and 9.88 ± 2.96 days, respectively (p-value = 0.0006; significant).

Time to resume routine activity was significantly less in the laparoscopically operated group than in the open surgery group. The mean duration to resume activity was 9.48 ± 2.29 days and 14.44 ± 5.03 days in the laparoscopy and open surgery groups, respectively (p-value = <0.0001; significant). Various characteristics evaluated in laparoscopic and open ventral hernia surgery are depicted in Table 6.

**Table 6:** A comparative analysis of parameters in laparoscopic and open ventral hernia surgery. *P-value of <0.05 is statistically significant. SD: standard deviation

Parameters	Laparoscopic group (Mean ± SD)	Open group (Mean ± SD)	t-value	P-value^*^
Defect size (cm)	2.54 ± 0.73	3.42 ± 1.81	2.25	0.029
Operative time (minutes)	57.52 ± 5.80	59.8 ± 11.15	0.906	0.369
Routine activity	9.48 ± 2.29	14.44 ± 5.03	4.483	<0.0001
Hospital stay (days)	7.4 ± 1.58	9.88 ± 2.96	3.693	0.0006

There were fewer postoperative complications among patients who underwent laparoscopic surgery compared to those who underwent open surgery (Table 7).

**Table 7:** Postoperative complications. *P-value of <0.05 is statistically significant. SSI: surgical site infection; ARDS: acute respiratory distress syndrome

Complications	Laparoscopic group (n = 25)	Open group (n = 25)	P-value*
Seroma	0	8 (32%)	0.004
Hematoma	0	0	-
SSI	1 (4%)	2 (8%)	1.000
Wound dehiscence	0	0	-
ARDS	1 (4%)	3 (12%)	0.609

Pain was more common in the open surgery group (88%) than in the laparoscopic group (64%) at the first follow-up (seven days after discharge) (p-value = 0.046; significant]. Chronic pain (six months post-surgery) was seen in two (8%) cases operated with open hernia repair and no case of laparoscopic repair. Recurrence was seen in one patient who was operated on following the open technique. A comparison with the literature is presented in Table 8.

**Table 8:** Comparison with the literature. LAP: laparoscopic group; OPEN: open group

Observation	Badiger et al. [8]		Basheer et al. [9]		Thota et al. [10]		Purushotham et al. [11]		Our study	
	LAP	OPEN	LAP	OPEN	LAP	OPEN	LAP	OPEN	LAP	OPEN
Patients	50	50	20	20	31	51	21	21	25	25
Operative time (minutes)	55	130	86	91	94.35	92.65	62.00 ± 5.84	38.05 ± 3.5	57.52 ± 5.80	59.8 ± 11.15
Infection rate (%)	0	2	5	15	3.2	17.6	0	9.5	4	8
Seroma rate (%)	3	8	30	10	6.4	41.1	4.8	4.8	0	32
Hospital stay (days)	2.6	6.8	1.15 ± 0.49	4.55 ± 4.14	4.64	15.17	1.37	3.56	7.4 ± 1.58	9.88 ± 2.96
Recurrence rate (%)	-	-	10	5	0	0	0	4.8	0	2

## Discussion

A ventral hernia is a common surgical problem with an increase in the repair rate annually. Ventral hernia incorporates a group of hernias that occur in the anterior abdominal wall, including incisional, umbilical, epigastric, and paraumbilical hernias [12]. Hernias are commonly managed by laparoscopic or open surgery, either by tissue repair or mesh repair [13].

Laparoscopic ventral hernia repair was started by LeBlanc in 1993. After that, evaluations were done to make laparoscopic hernia easier and safer for ventral hernia repair [8]. The laparoscopic approach avoids large incisions and drain placement and thus there is a reduction in wound-related complications [14,15].

In our study, 50 patients with ventral hernias were included and divided into laparoscopic and open hernia repair groups with 25 patients included in each group according to the patient’s preference for the procedure. The mean age of patients who underwent laparoscopic and open hernia repair was 52.29 ± 12.96 years and 50.78 ± 11.88 years, respectively. Overall, 54% of patients were males while 46% of patients were females. The male:female ratio was 1:1.08 and 1.5:1 in the laparoscopic and open hernia repair groups, respectively. Swelling was the most consistent chief complaint, present in 100% of cases. The most common diagnosis was paraumbilical hernia (34%), followed by epigastric (22%) and umbilical hernia (22%). The most common comorbidity was hypertension and type 2 diabetes mellitus.

Patients were subjected to surgery (laparoscopic or open repair) and compared in terms of operative time, postoperative pain (using the VAS score), hospital stay, return to routine activity, chronic pain, and recurrence.

There is an ongoing debate regarding the operative time of laparoscopic versus open surgery for ventral hernia repair. It has been stated that laparoscopic repair is less time-consuming as an open repair often requires extensive lateral dissection and flap creation which is time-consuming [9]. However, in our study, the mean operative time was 57.52 ± 5.80 minutes in the laparoscopic surgery group and 59.8 ± 11.15 minutes in the open surgery group (no statistical difference was found in the operative time). Basheer et al. reported a mean operative time of 86 minutes in laparoscopic surgery and 91 minutes in open surgery [9]. Thota et al. reported a mean operative time of 92.65 minutes and 94.35 minutes in open repair and laparoscopic repair, respectively (p-value = 0.443; not significant) [10].

Postoperative pain is the most frequent complaint in patients following hernia surgery. VAS is a common tool used for pain assessment in the postoperative period. In our study, postoperative pain was higher in the open group than in the laparoscopic group (p-value = 0.001). Purushotham et al. in a similar study found that postoperative pain (VAS score) was more in the open group compared to the laparoscopic group [11].

In the era of daycare surgery, it has become very important to have a minimum duration of hospitalization for the patient. In this study, we tried to keep the duration of hospital stay minimum. While comparing the laparoscopic hernia surgery group with the open hernia repair group, it was found that the median hospital stay was seven days in the laparoscopic surgery group and 10 days in the open surgery group, while the mean hospital stay was 7.4 ± 1.58 days and 9.88 ± 2.96 days in the laparoscopic and open surgery group, respectively (p-value = 0.0006; significant). Similar results have been reported in the studies conducted by Basheer et al. [9], Badiger et al. [8], Thota et al. [10], and Purushotham et al. [11].

In our study, time to resume routine activity was significantly less in the laparoscopically operated group than in the open surgery group (p-value = <0.0001; significant). Badiger et al. noted that the mean time to return to activity was 4.1556 ± 2.13 days in the laparoscopic group and 13.9811 ± 3.27 days in the open technique group [8]. Purushotham et al. showed that 70.29% in the laparoscopic group resumed activity on the 14th day (mean = 14.81) whereas 54.45% in the open group resumed activity on the 16th day (mean = 23.62) [11]. Thota et al. noted that the return to normal activity was 3.61 days in the laparoscopic surgery group and 29.7 days in the open surgery group [10].

Postoperative complications play a major role in a surgical patient and add to further morbidity of the patient. Although hernia surgery is considered to be a clean surgery, it is not completely free of complications. Hematoma, seroma, surgical site infections (SSIs), and wound dehiscence are among the common complications following hernia surgery.

In the present study, among all the patients, seroma was the most common complication which was noted in eight (16%) cases. SSI was seen in three (6%) cases and acute respiratory distress syndrome (ARDS) occurred in four (8%) cases. Postoperative complications were less in the laparoscopic repair group than in the open repair group, with the incidence of seroma more in the open surgery group and the difference being statistically significant (p-value = 0.004). Similar results have been noted in the studies conducted by Thota et al. [10] and Badiger et al. [8].

Persistent pain requiring prolonged analgesia use after hernia surgery is found to be distressing for both patients and the operating surgeon. While comparing laparoscopic and open hernia repair groups, we found that the incidence of chronic pain (lasting for more than six months) was more after open ventral hernia repair compared to laparoscopic repair.

Thota et al. also found chronic pain to be associated with patients who underwent open hernia surgery more compared with laparoscopic hernia surgery [10].

This was a single institution study with a small sample size. The period of assessment for recurrence rate was short (six months) which should have been longer for better assessment.

## Conclusions

Laparoscopic surgery for ventral hernia has become the preferred choice. Laparoscopic ventral hernia repair is better than open ventral hernia repair with regard to less postoperative pain, shorter hospital stay, faster return to normal routine activity, and low incidence of complications of surgery such as seroma and SSIs. A statistically significant difference was noted with regard to the above parameters in our study.
